# Draft genome assemblies of four manakins

**DOI:** 10.1038/s41597-022-01680-0

**Published:** 2022-09-13

**Authors:** Xuemei Li, Rongsheng Gao, Guangji Chen, Alivia Lee Price, Daniel Bilyeli Øksnebjerg, Peter Andrew Hosner, Yang Zhou, Guojie Zhang, Shaohong Feng

**Affiliations:** 1 College of Life Sciences, University of Chinese Academy of Sciences, Beijing, 100049 China; 2 BGI-Shenzhen, Shenzhen, 518083 China; 3 Villum Centre for Biodiversity Genomics, Section for Ecology and Evolution, Department of Biology, University of Copenhagen, Ole Maaløes Vej 5, 2200 Copenhagen, Denmark; 4 GLOBE Institute, Section for Evolutionary Genomics, University of Copenhagen, Copenhagen, Øster Farimagsgade 5, 1014 Copenhagen, Denmark; 5 Natural History Museum of Denmark, University of Copenhagen, Universitetsparken 15, 2100 Copenhagen, Denmark; 6 Villum Center for Global Mountain Biodiversity, Biodiversity Section, GLOBE Institute, University of Copenhagen, Universitetsparken 15, 2100 Copenhagen, Denmark; 7 Evolutionary & Organismal Biology Research Center, Zhejiang University School of Medicine, Hangzhou, 310058 China; 8 Liangzhu Laboratory, Zhejiang University Medical Center, 1369 West Wenyi Road, Hangzhou, 311121 China; 9 Innovation Center of Yangtze River Delta, Zhejiang University, Jiashan, 314102 China

**Keywords:** Evolution, Genome

## Abstract

Manakins are a family of small suboscine passerine birds characterized by their elaborate courtship displays, non-monogamous mating system, and sexual dimorphism. This family has served as a good model for the study of sexual selection. Here we present genome assemblies of four manakin species, including *Cryptopipo holochlora, Dixiphia pipra* (also known as *Pseudopipra pipra*), *Machaeropterus deliciosus* and *Masius chrysopterus*, generated by Single-tube Long Fragment Read (stLFR) technology. The assembled genome sizes ranged from 1.10 Gb to 1.19 Gb, with average scaffold N50 of 29 Mb and contig N50 of 169 Kb. On average, 12,055 protein-coding genes were annotated in the genomes, and 9.79% of the genomes were annotated as repetitive elements. We further identified 75 Mb of Z-linked sequences in manakins, containing 585 to 751 genes and an ~600 Kb pseudoautosomal region (PAR). One notable finding from these Z-linked sequences is that a possible Z-to-autosome/PAR reversal could have occurred in *M. chrysopterus*. These *de novo* genomes will contribute to a deeper understanding of evolutionary history and sexual selection in manakins.

## Abstract (summary)

**Table:** 

Measurement(s)	whole genome sequencing
Technology Type(s)	BGISEQ-500 Sequencing
Sample Characteristic - Organism	Pipridae

## Background & Summary

Manakins (Aves: Pipridae), a family of Passeriformes, contain 17 genera and about 50 species distributed across the Neotropics, and have some unique behavioral and morphological features^1^. Most species in the family have sexual dimorphism in plumage color^2^ and are polygynous^3,4^. Moreover, the complex courtship displays of males, which include high-speed movements, sophisticated acrobatics, coordinated movements of multiple males, mechanical and vocal sounds and constructed display site construction^5,6^, makes this lineage a fascinating model for studying sexual selection. During mating periods, males hold territories or aggregate for competitive displays to attract females for the chance to mate^7^. Courtship varies substantially among genera and species^8–11^. For example, in genus *Chiroxiphia*, one male forms a partnership with another male and they perform elaborate courtship dances and sing common songs together^12^. In contrast, *Corapipo gutturalis* does not cooperate with other males during courtship displays^13^. *Xenopipo atronitens* males elaborate courtship displays by making mechanical sounds through flapping their wings^14^, whereas *Lepidothrix coronata* males sing to attract females in addition to acrobatic displays^15^.

Courtship behavior plays an important role in attracting the opposite sex, increasing the chance of producing offspring and improving the reproductive rate of birds^2,16^. At present, the courtship display of manakin species has been studied from the aspect of behavior observation^17,18^, neuroendocrine^14,19,20^ and physiology^2^. The genetic mechanisms have also been discussed^21–24^, yet insights are lacking due to a lack of comparative genomic and transcriptomic data. As courtship displays are derived from sexual selection^25,26^, we expect that investigating the evolution of their genomes, particularly the sex chromosomes, could bring insights to the understanding of underlying genetic mechanisms. To address this knowledge gap, we conducted whole genome sequencing of four species representing four manakin genera: *C. holochlora, D. pipra, M. deliciosus* and *M. chrysopterus*^27,28^. Genome sizes of these four manakin species were estimated to be 1.15 Gb, the contig N50 ranged from 125 Kb to 212 Kb, and the scaffold N50 ranged from 18.4 Mb to 36.6 Mb. We annotated about 12,055 protein-coding genes on each manakin genome. On average, 99.97% of the predicted protein-coding genes were successfully annotated by three functional databases (SwissProt, InterPro, and KEGG). About 75 Mb of Z-linked sequences, including an ~600 Kb PAR, were identified from the available female manakin genomes, including two published species (*Corapipo altera* and *Neopelma chrysocephalum*). These genomic resources will benefit research on genetic mechanisms of manakin courtship displays, and other behavioral and ecological aspects.

## Methods

### Sample collection, library construction, and sequencing

Tissue samples of four manakin species (*C. holochlora, D. pipra, M. deliciosus* and *M. chrysopterus*) were provided by the Natural History Museum of Denmark. High-molecular-weight genomic DNA of these samples was extracted with the Kingfisher Cell and Tissue DNA Kit Protocol. Single tube-Long Fragment Read (stLFR) technology^29^ was used to construct the libraries for each sample. The resulting libraries underwent DNA Nanoball (DNB™) generation and DNBSEQ sequencing in 100 + 100 + 30 mode. On average, 149 Gb raw reads were produced for each species (Table 1).

**Table 1:** Sequencing reads statistics.

species		*Cryptopipo holochlora*	*Dixiphia pipra*	*Machaeropterus deliciosus*	*Masius chrysopterus*
Intitution acronym		NHMD	NHMD	NHMD	NHMD
SpecimenCode		B-126359	B-126493	B-125026	B-125031
Genus		*Cryptopipo*	*Dixiphia*	*Machaeropterus*	*Masius*
SpeciesName		*holochlora*	*pipra*	*deliciosus*	*chrysopterus*
subspecies		*holochlora*	*discolor*	/	*pax*
DateCollected		6-Jul-94	6-Jul-94	15-Apr-91	12-Sep-90
Sex		Not recorded	Not recorded	Not recorded	Not recorded
Field number		NK4-15.11.94	NK6-13.7.94	NK10-15.4.91	NK17-12.9.90
Sample in		EDTA	EDTA	EDTA	EDTA
voucher		skin; MECN:6936	Not recorded	skeleton; Salango Museum	skeleton; Museo Arqueológico Salango
CollectedBy		Niels Krabbe	Niels Krabbe	Niels Krabbe	Niels Krabbe
Location		Yasuní, Napo, Ecuador	Parque Nacional Yasuní, Napo, Ecuador	9 km west of Piñas, El Oro, Ecuador	Above Chinapinza, Zamora-Chinchipe, Ecuador
LocLatitude		−0.63333	−0.63333	−3.65	−4.039
LocLongitude		−76.43333	−76.43333	−79.75	−78.583
Elevation		300	300	900	1700
strategy		stlFR	stlFR	stlFR	stlFR
Sequencing platform		DNBseq	DNBseq	DNBseq	DNBseq
Library Insert Size (bp)		200~2000	200~2000	200~2000	200~2000
Raw reads	Total Data (Gb)	155.92	119.28	152.54	169.08
	Reads Length (bp)	100 + 100 + 30	100 + 100 + 30	100 + 100 + 30	100 + 100 + 30
	Sequence depth (×)	113	81	109	112
Clean reads	Total Data (Gb)	111.62	90	108.28	117.25
	Reads Length (bp)	100 + 100	100 + 100	100 + 100	100 + 100
	Sequence depth (×)	105	79	101	101

### Genome assembly and quality evaluation

A series of filtering steps was applied to these stLFR reads prior to the downstream analyses using SOAPfilter2 package (v2.2).

1. Remove reads with more than 10% of N bases;
2. Remove reads with more than 40% low quality bases (Phred score < = 10);
3. Remove reads with undersize insert size;
4. Filter out the PCR duplicates.

All cleaned stLFR library reads were transformed into 10X Genomics linked-reads format and passed into Supernova software (v2.0.1)^30^ to assemble the genome under the “pseudohap” mode for each species. After removing scaffolds with “N” >80%, GapCloser (v1.12)^31^ was used to close the intra-scaffold gaps.

**Fig. 1:**
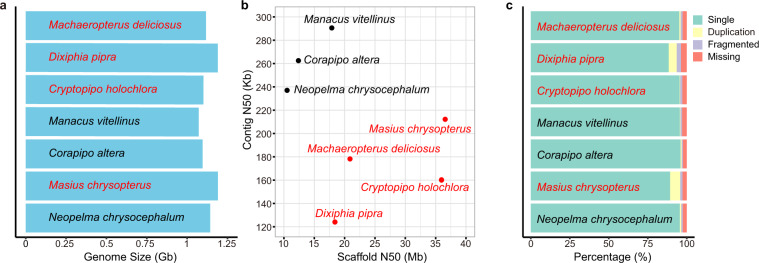
Genome assembly statistics of four manakin genomes assembled in this study and three previously published genomes. (**a**) Comparison of genome sizes. (**b**) Distribution of N50 statistics of the manakin genomes. Each dot represents a manakin species, with the x-axis representing the value of scaffold N50 and the y-axis representing the value of contig N50. (**c**) BUSCO analysis of the seven manakin genomes. Assembly completeness is shown as the percentage of single, duplicated, fragmented and missing genes. Four newly assembled manakin genomes in this study were marked in red, while three published ones in black. Three published species are *Corapipo altera* (GCF_003945725.1), *Manacus vitellinus* (GCF_001715985.3) and *Neopelma chrysocephalum* (GCF_003984885.1).

The size of the four assembled genomes are about 1.15 Gb, similar to the sizes of other avian genomes^32^ (Fig. 1a, Table 2). The scaffold N50 of all species is higher than 18 Mb, with the largest scaffold N50 found in *M. chrysopterus* (36 Mb). The contig N50 of all species is higher than 124 Kb. (Fig. 1b, Table 2).

**Table 2:** Genome assembly and BUSCO statistics.

Species	Genome assembly			BUSCO			
	Contig N50 (bp)	Scaffold N50 (bp)	Genome Size (bp)	Single (%)	Duplication (%)	Fragmented (%)	Missing (%)
*Corapipo altera*	262,501	12,385,833	1,095,745,976	96.10	0.60	0.90	2.40
*Cryptopipo holochlora*	160,303	35,963,530	1,099,400,137	95.40	0.30	1.40	2.90
*Dixiphia pipra*	124,585	18,411,073	1,187,686,032	88.40	5.10	2.80	3.70
*Machaeropterus deliciosus*	178,415	23,705,376	1,116,150,816	95.20	0.90	1.30	2.60
*Masius chrysopterus*	212,472	36,568,189	1,189,220,944	89.30	6.50	1.40	2.80
*Manacus vitellinus*	290,580	17,883,582	1,072,328,541	95.50	0.40	1.10	3.00
*Neopelma chrysocephalum*	237,029	10,517,223	1,142,796,179	95.50	0.90	1.00	2.60

We applied BUSCO (v5.2.2)^33^ to evaluate the completeness of these seven manakin genomes using aves_odb10 as the reference gene set. On average 92% of the core genes were assembled as complete single-copy genes in the four manakin genomes and only about 3% of the core genes could not be annotated on the four manakin genomes (Fig. 1c, Table 2). Therefore, the overall quality of the newly assembled genomes was high and comparable to other published manakin assemblies.

### Repeat annotation

Tandem repeats were identified by Tandem Repeat Finder (TRF, v4.09.1)^34^, and transposable elements (TEs) were annotated using a combination of homology-based RepeatMasker (v4.1.2)^35^, and *de novo* methods with RepeatModeler (v2.0.2a)^36^ and LTR_Finder(v1.07)^37^. The homology-based annotation of TEs was performed by RepeatMasker with its built-in library. RepeatModeler and LTR_Finder methods were used to build the *de novo* repeat library for each species, which was further used by RepeatMasker to predict repeats for each species.

**Fig. 2:**
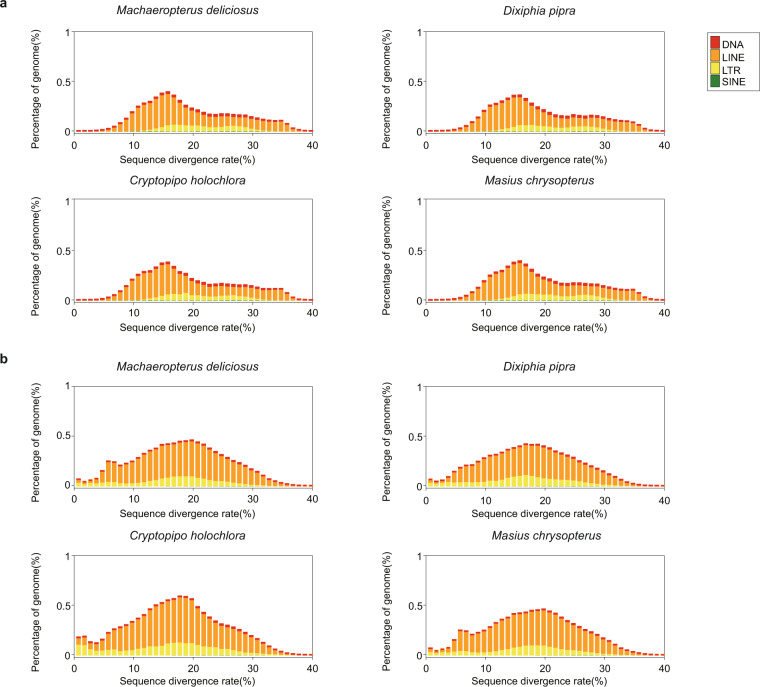
Distribution of divergence rate of four types of transposable elements (TEs) in the four manakin genomes. (**a**) The divergence rate was calculated between the identified TEs in the genome by homology-based method and the consensus sequence in the built-in RepeatMasker TE library. (**b**) The divergence rate was calculated between the identified TEs in the genome by *de novo* and the consensus sequence in the *de novo* TE library.

We found that the four species contained an average of 9.79% TEs in the genomes, with the proportions of each type being similar across these species (Fig. 2, Table 3). Long Interspersed Nuclear Elements (LINEs) accounted for most TEs, occupying about 6.79% of the genome.

**Table 3:** Repeats statistic.

species	DNA		LINE		SINE		LTR		Unknown		total	
	Length (bp)	% in genome	Length (bp)	% in genome	Length (bp)	% in genome	Length (bp)	% in genome	Length (bp)	% in genome	Length (bp)	% in genome
*Cryptopipo holochlora*	3,878,915	0.35	76,847,348	6.99	1,170,656	0.11	23,590,172	2.15	8,465,390	0.77	106,843,742	9.72
*Dixiphia pipra*	3,856,289	0.32	73,600,596	6.20	1,420,049	0.12	25,019,052	2.11	9,522,408	0.80	107,604,264	9.06
*Machaeropterus deliciosus*	3,354,142	0.30	77,377,782	6.93	1,362,779	0.12	35,341,855	3.17	8,143,000	0.73	118,332,726	10.60
*Masius chrysopterus*	3,596,970	0.30	83,835,605	7.05	1,338,816	0.11	23,337,458	1.96	10,145,405	0.85	116,163,527	9.77

### Protein-coding gene annotation

We applied the homolog-based approach to annotate the protein-coding genes by using the protein sequences of *Gallus gallus*, *Taeniopygia guttata* and *Homo sapiens* downloaded from Ensembl release 105 as the reference gene sets. The protein sequences of these reference genes were aligned to each genome using TBLASTN (v2.2.26)^38^ with an e-value cut off 1e-5, and multiple adjacent hits of the same query were connected by genBlastA (v1.0.4)^39^. Homologous blocks with length greater than 30% of the query protein length were retained. The connected hit region was later extended to include its 2 Kb upstream and downstream flanking regions, on which gene structure was predicted by Genewise (v2.4.1)^40^. MUSCLE (v3.8.31)^41^ was then used to align the annotated protein with the reference protein. Predicted proteins with length ≥30 amino acids and identity value ≥40% were retained. Pseudogenes (annotated genes containing >2 frame shifts or >1 premature stop codon) and retrogenes were further removed.

To build a non-redundant gene set, we first used hierarchical clustering^42^ to combine the homologous-based gene sets of *G*. *gallus* and *T. guttata*. The gene model with the highest identity to the query was preserved if a locus has been annotated with more than one gene model. By doing so, we obtained 8,250 protein-coding genes on average after removing the highly duplicated genes (genes had >10 duplicates, were single-exon genes, and overlapped with the repeats in >70% of coding region). In the end, the newly annotated loci from the human gene set, i.e., the gene model did not overlap with the above combined one, were added into the results. In summary, we predict an average of 12,055 protein-coding genes for each manakin with an average gene length of 22,952 bp. (Table 4).

**Table 4:** Protein-coding gene statistics.

species	# Total gene	# Single exon gene	Mean gene length (bp)	Mean coding sequence length (bp)	# Mean exons per gene	Mean exon length (bp)	Mean intron length (bp)
*Cryptopipo_holochlora*	11,681	702	22,859	1,677	9.89	170	2,384
*Dixiphia pipra*	11,770	703	22,821	1,669	9.84	170	2,393
*Machaeropterus_deliciosus*	11,985	730	22,880	1,689	9.91	170	2,378
*Masius_chrysopterus*	12,785	761	23,247	1,685	9.88	171	2,428

### Gene function annotation

The translated gene coding sequences were aligned to the SwissProt database (release-2020_05)^43^ using BLASTP (v2.2.26)^38^ with e-value cutoff 1e-5. The best match was assigned as the function annotation for each gene. Motifs and domains of each gene was annotated with modules PRINTS, SMART, PANTHER, ProSiteProfiles, ProSitePatterns, CDD, SFLD, Gene3D, SUPERFAMILY, and TMHMM of InterPro (v5.52–86.0)^44^. To identify the pathways in which genes may be involved, we also aligned the protein sequence of each gene to the KEGG database (release-93)^45^ using BLASTP (v2.2.26)^38^ with e-value cutoff 1e-5. Overall, 99.97% of the protein-coding genes of the four manakin genomes were annotated by the functional databases (Table 5).

**Table 5:** Function annotation results.

species	Swissprot		KEGG		Interpro		Overall	
	#gene	%	#gene	%	#gene	%	#gene	%
*Corapipo altera*	15,772	96.29	14,835	90.57	15,858	96.81	15,987	97.60
*Cryptopipo holochlora*	11,652	99.75	10,972	93.93	11,669	99.90	11,677	99.97
*Dixiphia pipra*	11,742	99.76	11,036	93.76	11,762	99.93	11,768	99.98
*Machaeropterus deliciosus*	11,958	99.75	11,276	94.07	11,980	99.93	11,985	99.97
*Manacus vitellinus*	14,204	98.03	13,299	91.79	14,254	98.38	14,317	98.81
*Masius chrysopterus*	12,754	99.76	12,027	94.07	12,770	99.88	12,781	99.97
*Neopelma chrysocephalum*	16,229	96.01	15,287	90.44	16,302	96.44	16,447	97.30

### Orthology assignment and phylogeny inference

To reconstruct the phylogenetic history of the seven genera in manakins, we chose one representative species for each genus, including the four species in this study and three published species (*C. altera*: GCF_003945725.1, *M. vitellinus*: GCF_001715985.3 and *N. chrysocephalum*: GCF_003984885.1). *T. guttat*a (GCF_003957565.2) and *Calypte. anna* (GCF_003957555.1) were used as outgroups. The protein-coding gene sets of these species were obtained from NCBI. We used the *T. guttata* gene sets as the reference and performed a BLASTP (v2.2.26)^38^ search on the protein sequences with an e-value cut-off of 1e-5. The reciprocal best hit (RBH) orthologs between *T. guttata* and every other species were identified following the published literature^46^ but without the evidence of genomic synteny. In total, we obtained 9,654 one-to-one orthologs of these nine species by merging pairwise orthologs according to the reference *T. guttata* gene set.

**Fig. 3:**
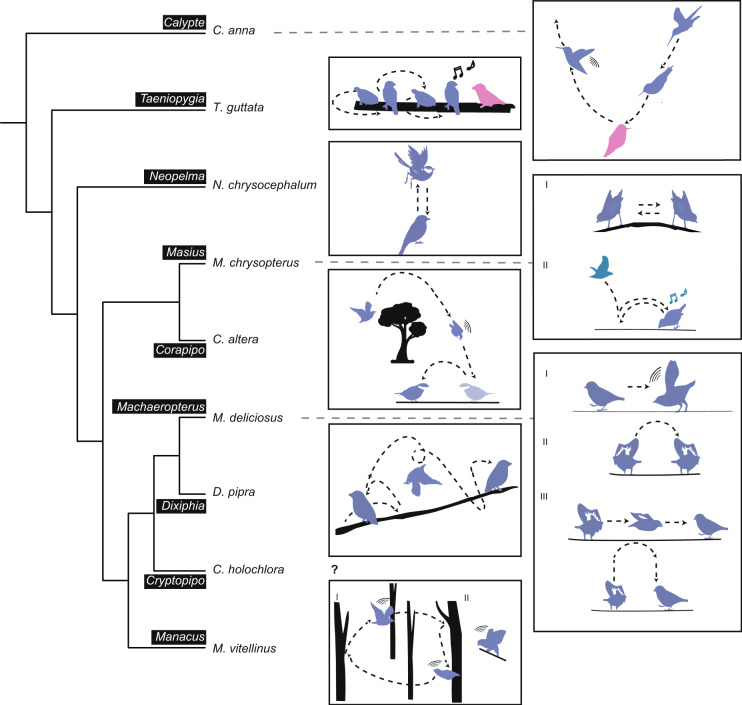
Species tree with courtship displays. The ASTRAL species tree has the local posterior probabilities of all branch support as 1.0 across the tree. *C. anna*: The male ascends and swoops over the female. As the male nears the bottom of the dive, it flies upwards and its tail feathers make a sound^90^. *T. guttata*: The male jumps in the direction of the female, rotating 180° with each hop, moving its head and tail, and singing. When facing a female, the male sings and rhythmically shakes its head^91^. *N. chrysocephalum*: The male flaps its wings in a vertical leap^92^. *M. chrysopterus*: I. The male performs a side-to-side bow, with his head down and his tail up, turning his body 90°–180° degrees as he bows. II. The male flies to the log, then jumps to another place, lands on the log and sings. This can be done by two males working together^93^. *C. altera*: The male flies up from the display log, following by a high speed descent, wings making a sound, turns in the air, and lands facing the original landing site^94^. *M. deliciosus*: I. The male produces mechanical sounds by flapping their wings. II. When the male stands perpendicular to the perch, he bends forward, jumps from side to side, as if to display the black and white markings on the wings. but makes no sound. III. The male first flies along the perch in a short distance and then flies vertically upward, turning its body 180° in the process^95^. *D. pipra*: The male low jumped forward and high jumped back, spins his body in the air in a somersault-like motion, then flies to the perch and lands on it^10^. *C. holochlora*: We don’t have much information about its courtship. *M. vitellinus*: The male performs snap-jump displays, jumping from one sapling to another, shaking its wings in midair. II. The male flaps its wings to produce mechanical sounds^96^. In the silhouette males are in blue and females are in pink.

**Fig. 4:**
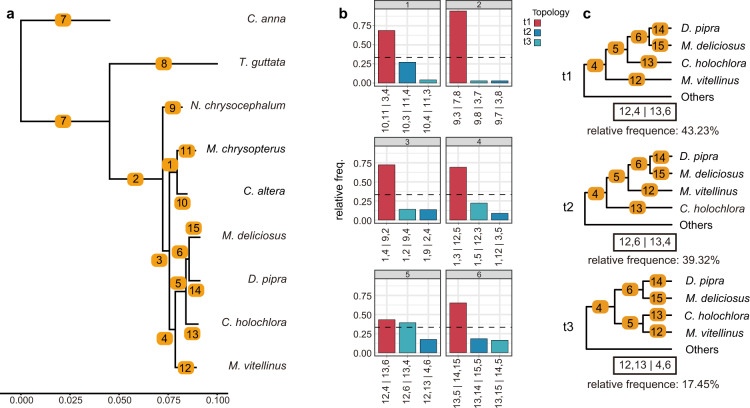
Discordance of gene trees with the species tree. (**a**) The branch lengths are estimated by RAxML based on the concatenated ortholog alignments. The branch length scale refers to substitutions per site. (**b**) Frequency of three potential topologies around focal internal branches of the ASTRAL species tree. Main topology (species tree) is shown in red, and the other two alternative topologies are shown in light and dark blue. The dotted line indicates the 1/3 threshold. The title of each subfigure indicates the label of the corresponding branch on the tree in panel a. Each internal branch has four neighboring branches which could be used to represent quartet topologies. On the x-axis the exact definition of each quartet topology is shown using the neighboring branch labels separated by “|”. (**c**) Example of the three topologies with the relative frequency values for internal branch 5, corresponding to panel 5 in b. The alternative topologies for other branches can be found in the Figshare database^89^.

The phylogeny of nine species was inferred based on the coalescent-based method, ASTRAL-III (v5.14.2)^47^. First, ortholog alignments were generated as follows: (1) we aligned the protein sequences with MAFFT L-INS-I (v7.487)^48^; (2) we used trimAl (v1.4.rev15)^49^ to achieve a column-based alignment filtering with the parameter “automated”, i.e., a heuristic selection of the automatic method based on similarity statistics; and (3) the nucleic acid alignments were back-translated from the trimmed protein alignments. After these steps, we obtained 9,653 trimmed ortholog alignments containing 805,481 parsimony informative sites in total. Then, we inferred the gene tree for each ortholog alignment using IQ-TREE (v1.6.12)^50^ with ModelFinder^51^ function to determine the best-fit model. The output gene trees were next used as the input for ASTRAL-III (v5.14.2)^47^ with default parameters to infer the species tree shown in Fig. 3. As ASTRAL-III measures the branch lengths in coalescent units, we further ran RAxML (v8.2.12)^52^ under GTR + GAMMA substitution model to estimate the branch lengths in substitution per site for the concatenated ortholog alignments by specifying the ASTRAL species tree (Fig. 4a). We also used DiscoVista^53^ to analyze the discordance frequencies between the ASTRAL species tree and the 9,653 gene trees (Fig. 4b). The frequency of three potential topologies is inferred based on the focal internal branches of the species tree with the main topology (in red) and alternative topologies (in blues). More phylogenetic discordance can be observed in branch 5. Specifically, the frequency of the gene trees that support *C. holochlora* or *M. vitellinus* as the sister clade to *D. pipra* and *M. deliciosu*s is close (Fig. 4c). In contrast to our species tree based on the coding regions, the UCEs-based topology published by Leite *et al*. 2021 concluded *M. vitellinus* as the sister clade to *D. pipra* and *M. deliciosu*s^54^. Previous studies have suggested that such topological differences could result from data-type effects^55,56^. As in Leite *et al*. 2021 study, the UCE-based and exon-based topologies were not consistent either. Considering that our result still differed from their reported tree even based on coding regions, we assumed that such conflicts of *C. holochlora* and *M. vitellinus* could be caused by their limited parsimony informative sites, our restricted number of species, or the evolutionary forces (e.g. introgression and incomplete lineage sorting). More whole genome resources are needed to solve the phylogenomics of these genera.

### Selection analysis of plumage color related genes

Manakins are characterized by a variety of plumage colors^57–59^. To explore the possible genetic mechanism of the color diversity, we investigated the signatures of selection on 37 orthologous genes related to plumage color reported in previous studies^60–69^. With our phylogenetic tree, the maximum likelihood estimation of *dN* (non-synonymous substitution rate), *dS* (synonymous substitution rate), and ω (*dN*/*dS*) for each gene was performed under two branch models, one-ratio model (H0) and free-ratio model (H1), by using codeml program in PAML package (v4.9)^70^. Likelihood ratio test was used to test if H1 was significantly better than H0, and the output p-values were next corrected with the false-discovery rate (FDR) method. Under FDR-corrected p-value cutoff 0.05, if a branch showed ω > 1 in the branch model analysis, the gene was considered to be positively selected at this branch. We further filtered results with abnormally high ω values (ω > 3)^71^. We finally obtained four genes, *TBC1D22A*, *EDA*, *SLC45A2* and *GOLGB1*, that were likely to have undergone positive selection during manakin evolution. Among them, *SLC45A2* was found to be positively selected in *M. deliciosus*. The gene encodes a transporter protein that mediates melanin synthesis^66^. As pheomelanin is responsible for brown and reddish coloration^72,73^, the positive selection signal in *M. deliciosus* may explain its unique reddish-brown body plumage among other studied manakin species. The other three genes were found under positive selection in the internal branches. *TBC1D22A* was positively selected in the most recent common ancestor (MRCA) of *M. deliciosus, D. pipra* and *C. holochlora* (branch 5 in Fig. 4a). *EDA* was positively selected in the MRCA of *M. deliciosus, D. pipra, C. holochlora* and *M. vitellinus* (branch 4 in Fig. 4a). *GOLGB1* was positively selected in both *M. chrysopterus* and MRCA of *M. deliciosus, D. pipra* and *C. holochlora* (branch 5 in Fig. 4a).

### Sex chromosomes

Unlike mammals where males are heterogametic (XY system), in birds the females are heterogametic (ZW system). The avian ZW chromosomes are evolved from a pair of ancestral autosomes about 102 million years ago^74^. During evolution, the differentiation of sex chromosomes is caused by recombination arrests on the W chromosome, resulting in the reduction of functional genes on the chromosome and the accumulation of repetitive elements. The Z and W chromosomes of the extant Neoaves are of great differences in length and gene content^74^. Only a small PAR remains for recombination during cell division in females^74^.

**Fig. 5:**
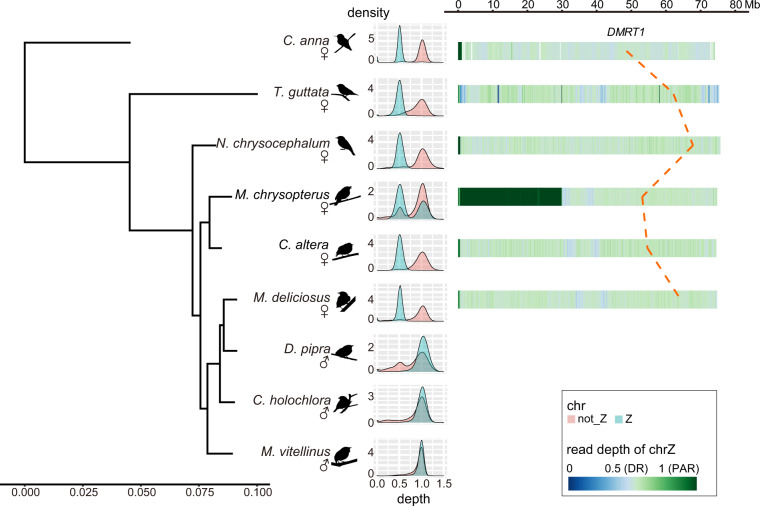
Sex-related information of manakins. The sex of each sequenced bird is confirmed with the sequencing depth distribution of candidate Z (blue) and other sequence (not_Z, pink) shown in the middle. The putative Z chromosomes of species with a female sequenced are shown on the right. Each female avian species has a color-coded track showing the female read depth of chrZ under 100 Kb resolution, where PAR is shown in dark green, DR in light green and assembly gaps as blanks. One exception is found in *M. chrysopterus* where a large region shows normalized female sequencing depth around one, implying that a DR-to-autosome/PAR reversal might have happened in this species. The positions of the putative sex-determining gene *DMRT1* were traced with the dotted red line.

We first confirmed the sex of the manakin samples by mapping the sequencing reads of the same individual to its genomes with BWA MEM (v0.7.17)^75^. Coverage information extracted by samtools (v1.9)^76^ was calculated in 5 Kb non-overlapping windows with bedtools (v2.29.2)^77^ and normalized by the peak coverage. We also softmasked the genomes and performed LASTZ(v1.04.00)^78^ alignment with the manakin genomes using the *T. guttata* genome as a reference with parameter set ‘--step = 19 --hspthresh = 2200 --inner = 2000 --ydrop = 3400 --gappedthresh = 10000 --format = axt’. Based on the assumption that Z chromosomes are relatively conserved among avians, scaffolds mapped to the Z chromosome of *T. guttata* with the aligning rate >50% were treated as candidate Z-linked sequences. The distribution of normalized coverage of candidate Z and other (not_Z) sequences were then visualized to check the sex of the sequenced individuals. We confirmed most of the sex information was consistent with records except *M. vitellinus* (BioSample SAMN02299332). This sample is more likely to be a male instead of a female. Its normalized coverage distribution was similar between the Z and not_Z sequences, with both peaks at around one and without a rise at 0.5 (Fig. 5).

With the above procedures we identified about 75 Mb of Z-linked scaffolds containing 585 to 751 genes in the manakins species where a female was sequenced (Fig. 5, Table 6, Supplementary Tables 1 and 2). We further constructed these Z-linked sequences into pseudo-Z-chromosomes for visualization with Ragtag (v2.1.0)^79^ using *T. guttata* Z chromosomes as reference under parameter set “-q 10 -d 100,000 -i 0.2 -a 0.0 -s 0.0 -g 100 -m 100000 –aligner minimap2 –mm2-params ‘-x asm5’”. To obtain the genomic coordinate of the avian candidate sex determining gene *DMRT1*^80^, we used the *DMRT1* protein sequence of *G. gallus* downloaded from UniPort as a query, and annotated the orthologous genes on the pseudo-Z-chromosome of manakins using Genewise.

**Table 6:** Z chromosome statistics.

species	PAR candidate		chrZ (PAR included)	
	length (bp)	# gene	length (bp)	# gene
*Corapipo altera*	674,217	16	74,797,643	744
*Machaeropterus deliciosus*	610,563	11	75,476,763	585
*Masius chrysopterus*	29,966,716*	228	74,940,613*	602
*Neopelma chrysocephalum*	660,109	17	76,014,776	751
*Calypte anna*	1,095,000	20	74,081,004	703
*Taeniopygia Guttata*	495,000	17	75,396,176	802

*We suggest a differentiated region-to-autosome/pseudoautosomal region reversal has happened in this species.

We also used the normalized coverage to identify PAR in the genomes assembled from female individuals. Z-linked scaffolds with normalized depth greater than 0.7 were identified as PAR candidates. We found that PAR is conserved between manakins and *T. guttata* with length of about 600 Kb and containing about 16 genes. However, one exception was found in *M. chrysopterus* where the candidate PAR is 30 Mb and contains 228 genes (Fig. 5, Table 6 & Supplementary Table 1). Most of the 30 Mb region has become differentiated region (DR) in the most recent common ancestor of Neoaves for about 69 million years^74^, as well as the other manakins in this study. Thus, it is more likely that the region has reverted back to PAR or even autosome in *M. chrysopterus*. Such reversal is rare but has been found in other species^81,82^. Further exploration is required for the mechanism and explanation of this possible reversal.

## Data Records

The genome sequencing data and assembly of the four manakin species has been deposited to CNSA (https://db.cngb.org/cnsa/) of CNGBdb^83^ with accession number CNP0002887. The raw reads from DNBSEQ sequencing and the genome assembly of four manakins in this study was deposited to NCBI with SRA accession SRR19721507, SRR19721508, SRR19721509, SRR19721510, SRR19721511^84–88^. The annotation results of four manakin species, phylogenetic tree, discordance trees and the diploid assemblies were deposited in Figshare database^89^.

## Technical Validation

The assemblies of four manakins used in this study are the first version of the species. The average length of scaffold N50 and contig N50 were 29 Mb and 169 Kb, respectively. BUSCO analysis evaluated the genome assembly completeness. In total, about 95.23% core genes were assembled as complete genes of the four manakin genomes (single ~92.075%, duplicated ~3.200%, fragmented ~1.725%, missing ~ 3.000%). These results are comparable to those of three previously published manakins (*Corapipo altera*, *Manacus vitellinus*, and *Neopelma chrysocephalum*).

## Supplementary information

Supplementary Table 1

Supplementary Table 2

## Data Availability

The version and parameters of bioinformatic tools used in this study have been described in the Method section. If no parameter is described, the default is used.
